# Diversity and assembly mechanisms of zooplankton communities in freshwater aquaculture ponds

**DOI:** 10.1007/s42995-025-00297-7

**Published:** 2025-08-20

**Authors:** Chengzhi Mao, Xinghao Li, Micah Dunthorn, Wenxin Xu, Xiaotian Luo, Xueping Xiong, Saleh A. Al-Farraj, Jie Huang

**Affiliations:** 1 State Key Laboratory of Lake and Watershed Science for Water Security, Institute of Hydrobiology, Chinese Academy of Sciences, Wuhan, 430072 China; 2 Key Laboratory of Regional Development and Environmental Response, Hubei Engineering Research Center for Rural Drinking Water Security, Hubei University, Wuhan, 430062 China; 3 Natural History Museum, University of Oslo, 0318 Oslo, Norway; 4 Zoology Department, College of Science, King Saud University, 11451 Riyadh, Saudi Arabia

**Keywords:** Zooplankton, Community assembly, Ecological process, Freshwater aquaculture

## Abstract

Ecological succession is vital for forecasting ecosystem responses to environmental changes and their future states. Zooplankton, a primary natural food source in aquaculture, plays a crucial role in maintaining ecosystem function. Thus, understanding how zooplankton communities respond to environmental changes is essential for economic and ecological outcomes. In this study, we examined three types of aquaculture ponds (crab, crayfish, and fish ponds) with over 10 years of history and analyzed 27 environmental factors potentially influencing zooplankton dynamics throughout the year. Our results showed that Rotifera was the most abundant group in all three pond types, followed by Protista, Cladocera, and Copepoda. The dominant species across different seasons and ponds were *Polyarthra vulgaris*, *Anuraeopsis fissa*, and *Trichocerca pusilla*. The alpha diversity of zooplankton was influenced by various environmental factors across different pond types, with significant effects of antibiotics observed only in the fish ponds. The temporal and spatial distributions of zooplankton communities varied significantly. Deterministic processes, driven primarily by temperature and ammonia nitrogen, were identified as the primary mechanisms influencing zooplankton community assembly in freshwater aquaculture ponds. These findings inform management practices aimed at regulating key environmental drivers and optimizing zooplankton dynamics, with implications for maintaining ecosystem stability and productivity and, ultimately, supporting sustainable aquaculture.

**Supplementary Information:**

The online version contains supplementary material available at 10.1007/s42995-025-00297-7.

## Introduction

Freshwater aquaculture is a vital component of the global food supply. In China, pond farming dominates freshwater aquaculture, accounting for over 50% of freshwater aquaculture areas and contributing more than 70% of the total inland aquaculture production (Cao et al. 2007; Wang et al. 2015; China Fishery Statistic Yearbook 2021). However, significant concerns have been raised regarding the environmental impacts of freshwater aquaculture, particularly on aquatic ecosystems, which are directly affected by aquaculture activities. Although impacts on terrestrial ecosystems, such as nutrient runoff and land-use changes, are also possible, the most significant effects are on aquatic environments (Salin and Arome Ataguba 2018). Intensive aquaculture systems release large quantities of nutrients (e.g., nitrogen and phosphorus) and heavy metals and antibiotics into surrounding water bodies (Bian et al. 2012; Chen et al. 2020; Nemati et al. 2009). The overuse of fertilizers, feeds, and antibiotics can lead to eutrophication and water quality degradation and adversely affect biological communities in aquatic systems (Boyd 2018; Cao et al. 2007).

Zooplankton plays a critical role in maintaining aquatic ecosystem health by facilitating nutrient cycling and acting as a key link in aquatic food webs (Spoljar et al. 2016). It serves as a vital live feed for aquaculture species, holding significant economic value in the advancement of aquaculture (Lomartire et al. 2021; Lupatsch et al. 2001). The quality and quantity of this feed directly impact the growth of aquaculture species, indirectly influencing their maturation and mortality rates (Wootton 1990). Thus, effective understanding and management of zooplankton populations are crucial for the growth and survival of cultured organisms, such as fish and crustaceans (Mischke et al. 2019). Further, zooplankton are known to improve water quality by grazing on phytoplankton, including harmful cyanobacteria (Belfiore et al. 2021; Chislock et al. 2019; Triest et al. 2016). Understanding the diversity and dynamics of zooplankton communities in response to aquaculture activities is thus essential for assessing water quality and overall ecosystem function (Gannon and Stemberger 1978).

However, despite the recognized ecological importance of zooplankton, there is limited knowledge regarding how zooplankton communities respond to various aquaculture practices and environmental conditions. Specifically, research on how zooplankton communities are affected by seasonal variations, different cultured species, and varying farming practices is lacking. Previous studies have primarily focused on spatial and temporal changes in species composition and the correlation of zooplankton communities with environmental factors in specific types of aquaculture ponds or aquaculture-influenced lakes and reservoirs (Luo et al. 2020; Nguyen et al. 2020; Tulsankar et al. 2021). Temporal changes are often found to outweigh the effects of aquaculture-related factors, such as feed composition and pond age, in shaping zooplankton communities (Toth et al. 2020; Tulsankar et al. 2021), as has been observed in bacterial communities (Marmen et al. 2021; Zeng et al. 2019). Given the lack of comprehensive data on how these variables influence zooplankton diversity and dynamics, further research is necessary to bridge this gap and enhance our understanding of the interactions between aquaculture practices and zooplankton communities.

A widely recognized concept in microbial ecology posits that microbial community assembly is governed by both deterministic and stochastic processes (Meybeck 2003; Vass et al. 2020). Deterministic factors, such as abiotic environmental conditions (e.g., pH, salinity, temperature, and nutrients) and species interactions (e.g., competition, predation, and mutualisms), serve as strong selective forces that govern community composition (Liu et al. 2019; Nemergut et al. 2013). Meanwhile, stochastic processes, such as random changes in birth, death, speciation, and immigration, can also shape community patterns (Zhou and Ning 2017). The relative importance of deterministic and stochastic processes in shaping community structures remains debated. For instance, sediment resuspension in grass carp culture ponds has been shown to enhance deterministic processes by promoting homogeneous selection and reduce the dispersal limitation of protist communities (Zheng et al. 2021). In contrast, stochastic processes dominate in bacterial communities in shrimp ponds, reservoirs, and wastewater treatment plants (Ding et al. 2021; Hou et al. 2021). Moreover, distinct differences in species compositions and community structures, as well as variations in ecological processes, have been observed in planktonic and sedimentary bacterial communities in aquaculture-influenced and less-impacted lakes (Qin et al. 2020).

Deterministic and stochastic processes have also been described for plants and vertebrates, with the concepts of limiting similarity and environmental filtering explaining how abiotic and biotic factors constrain species composition. Limiting similarity suggests that species with similar ecological niches cannot coexist because of competitive exclusion, whereas environmental filtering posits that only species capable of tolerating specific environmental conditions can persist in a given habitat (Diamond 1975; MacArthur and Levins 1967). While these concepts have been foundational in explaining community assembly, recent research has emphasized the complexity of community dynamics, incorporating factors like historical contingency and dispersal limitations (David et al. 2021). These processes have been well studied in plants, vertebrates, and microbial systems (Chen et al. 2022; David et al. 2021; Li et al. 2025; Tang et al. 2020; Xing et al. 2021), but much less is known about how they govern zooplankton communities (Li et al. 2019).

To address this gap, we investigated the seasonal variations of zooplankton communities in three different aquaculture pond systems—crab, crayfish, and fish ponds—located in Honghu City, the largest freshwater aquaculture hub in China. These ponds vary in terms of the species cultured, pond management practices (e.g., water exchange rates, feeding regimes, and chemical treatments), and the overall intensity of aquaculture activities (e.g., stocking density and nutrient input). These factors are expected to influence the composition and dynamics of zooplankton communities. We hypothesized that differences in aquaculture practices, such as the type of species cultured and pond management strategies, would lead to distinct seasonal patterns in zooplankton communities across pond systems. Specifically, we expected these variations to result in differences in the seasonal dynamics of zooplankton. We aimed to explore the following questions: (1) Do zooplankton communities in different ponds follow distinct seasonal patterns, or are there common trends across pond types? (2) How do different aquaculture practices influence the community assembly processes of zooplankton? (3) What are the key factors driving zooplankton diversity patterns and community assembly mechanisms in freshwater aquaculture ponds?

## Materials and methods

### Study area, sample collection, and species identification

**Fig. 1:**
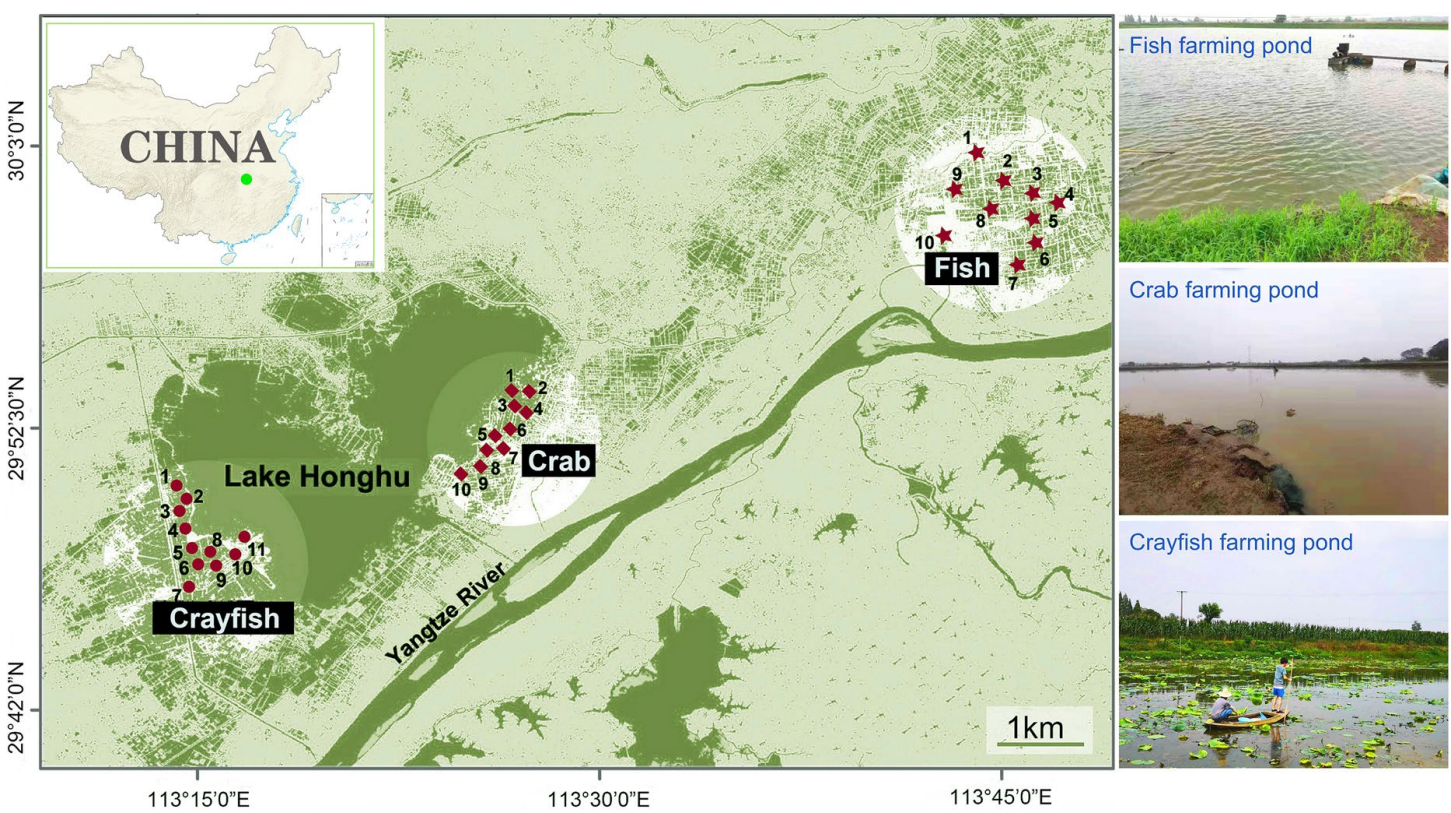
Location of the sampling sites in crab, crayfish, and fish ponds in Honghu Lake, China

This study investigated aquaculture ponds around Lake Honghu, the largest freshwater aquaculture area in China, known for its diverse types of aquaculture ponds. From April 2019 to January 2020, we conducted seasonal sampling (April, July, October, and January) from three typical types of ponds in the region: ten crab ponds (*Eriocheir sinensis*), eleven crayfish ponds (*Procambarus clarkii*), and ten fish ponds (*Ctenopharyngodon idellus*) (Fig. 1). The area of the investigated ponds ranged from 1.5 to 2 ha, with an average depth of 1–2 m. Sampling was conducted throughout the aquaculture cycle, from juvenile organisms (fry) to marketable size, after which the species were harvested and sold. The ponds were generally fertilized to enhance productivity, with regular feeding and water management to ensure optimal conditions. Water treatment agents, including disinfectants, algaecides, and antimicrobial agents, were applied periodically to maintain water quality and prevent diseases. The fish ponds typically required more frequent antimicrobial treatments, whereas the crayfish and crab ponds used algaecides occasionally to control algae growth. Harvest frequency varied by species and market demand, typically occurring once or twice a year. There were more frequent harvests for species with shorter life cycles, such as crayfish. The average yield per hectare was approximately 15,000–20,000 kg for fish ponds, approximately 1500 kg for crayfish ponds, and between 1500 and 2000 kg for crab ponds.

A total of 116 samples were collected: 36 from the crab ponds, 41 from the crayfish ponds, and 39 from the fish ponds. Partial samples could not be collected because of restrictive conditions, such as drained ponds or boat unavailability. Surface water samples were collected from each pond using a diagonal mix method, as described previously (Mao et al. 2025). Water samples were filtered through a 20-μm plankton net and preserved using 5% formalin and 10% Lugol’s solution for microscopic examination. Zooplankton was classified to the lowest practical taxonomic level, with morphological identification based on published references and documents (Koste 1978; Shen 1979; Yang et al. 2015; Zhou and Chen 2011). Biomass estimation was performed using abundance data and the average biomass of individuals, applying geometric methods for conversion (Filstrup et al. 2014; Huang 1982).

### Measurement of environmental variables

Regular water quality parameters such as temperature (Temp), pH, and dissolved oxygen (DO) were measured using a portable YSI 3074 carrying case (YSI, Ohio, USA). Concentrations of ammonium nitrogen (NH_4_^+^-N), nitrite nitrogen (NO_2_^−^-N), nitrate nitrogen (NO_3_^−^-N), total nitrogen (TN), total phosphorus (TP), and dissolved phosphorus (DP) were determined following the method of Bai et al. (2023). Total organic carbon (TOC) was detected using a MultiN/C 3100 analyzer (Analytik Jena, Germany). Heavy metals, including cadmium (Cd), zinc (Zn), copper (Cu), lead (Pb), chromium (Cr), arsenic (As), and selenium (Se), were measured using an inductively coupled plasma–mass spectrometer (NexION300X, PerkinElmer, MA) by Wang et al. (2022). Additionally, ten antibiotics—trimethoprim (TMP), sulfadiazine (SDZ), sulfamethoxazole (SMX), sulfamethazine (SMZ), enrofloxacin (EFX), doxycycline (DC), oxytetracycline (OTC), erythromycin (ETM), florfenicol (FF) and roxithromycin (RTM)—were analyzed and documented by Mai et al. (2023). Detailed physicochemical factors at each sampling site are provided in Table S1. 

### Alpha and beta diversity analysis

Venn diagrams were generated using an online tool (http://jvenn.toulouse.inra.fr/app/example.html). The richness and Shannon indices were calculated using the “vegan” and “MicrobiotaProcess” packages in R software (version 4.0.3) to evaluate the alpha diversity of zooplankton communities (Dixon 2003; Xu et al. 2023). Constrained principal coordinates analysis (CPCoA) based on the Bray–Curtis similarity was used to characterize the beta diversity of the zooplankton communities. Permutational multivariate analysis of variance (Adonis) and analysis of similarity (ANOSIM) were employed to test the significant differences in zooplankton communities among different seasons or between different ponds (Hu et al. 2017). 

### Correlating zooplankton communities with environmental factors

Pearson’s correlation analysis was conducted to assess the relationships between zooplankton community diversity and environmental factors. Canonical analysis of principal coordinates (CAP) was performed to evaluate the importance of environmental factors in community assembly using the “ordiR2step” function in the “vegan” package in R software (version 4.0.3). Standard and partial mantel tests were applied to evaluate the influence of environmental variables on zooplankton community structures using the “ecodist” package in R. The random forest package was used to construct a regression model to evaluate the relative importance of environmental factors on the distribution of zooplankton communities. Additionally, we identified the species most sensitive to seasonal changes in aquaculture ponds through random forest analysis (Breiman 2001; Trivedi et al. 2016). To estimate the sensitivity of zooplankton to seasonal changes, we calculated the percentage increase in the mean squared error (MSE) of variables. The “A3” package was used to obtain the significance and cross-validated *R*^2^ of the random forest model, with 999 permutations of the response variable (Jiao et al. 2018).

### Neutral and null community model analysis

The Sloan neutral model was used to assess the effects of stochastic dispersal on the assembly of zooplankton communities (Sloan et al. 2006). This model predicts the relationship between the occurrence frequency of species and their relative abundance across the broader metacommunity using a single free parameter that describes the migration rate “*m*” of individual zooplankton (Burns et al. 2016). The *R*^2^ value indicates the goodness of fit to the neutral model, and 95% confidence intervals around all fitting statistics were obtained through bootstrapping with 1000 bootstrap replicates (Sloan et al. 2006). In addition, we adopted the null model analysis improved by Chase et al. (2011), originally proposed by Raup and Crick (1979), in evaluating the relative importance of deterministic and stochastic processes in microbial community assembly (Chase et al. 2011; Raup and Crick 1979). The null model, based on the Raup–Crick dissimilarity index, establishes a range of variation between − 1 and 1. We assessed whether community compositions were significantly different by comparing the Raup–Crick index to the null expectation. A Raup–Crick index closer to 1 or − 1 suggests fewer or more species than expected by random chance, respectively. Furthermore, we used nonmetric multidimensional scaling (NMDS) based on the improved Raup–Crick dissimilarity index to visualize the similarity or dissimilarity between different communities (Chase 2007; Chase et al. 2011).

## Results

### Distribution patterns and seasonal variation of zooplankton communities in different aquaculture ponds

**Fig. 2:**
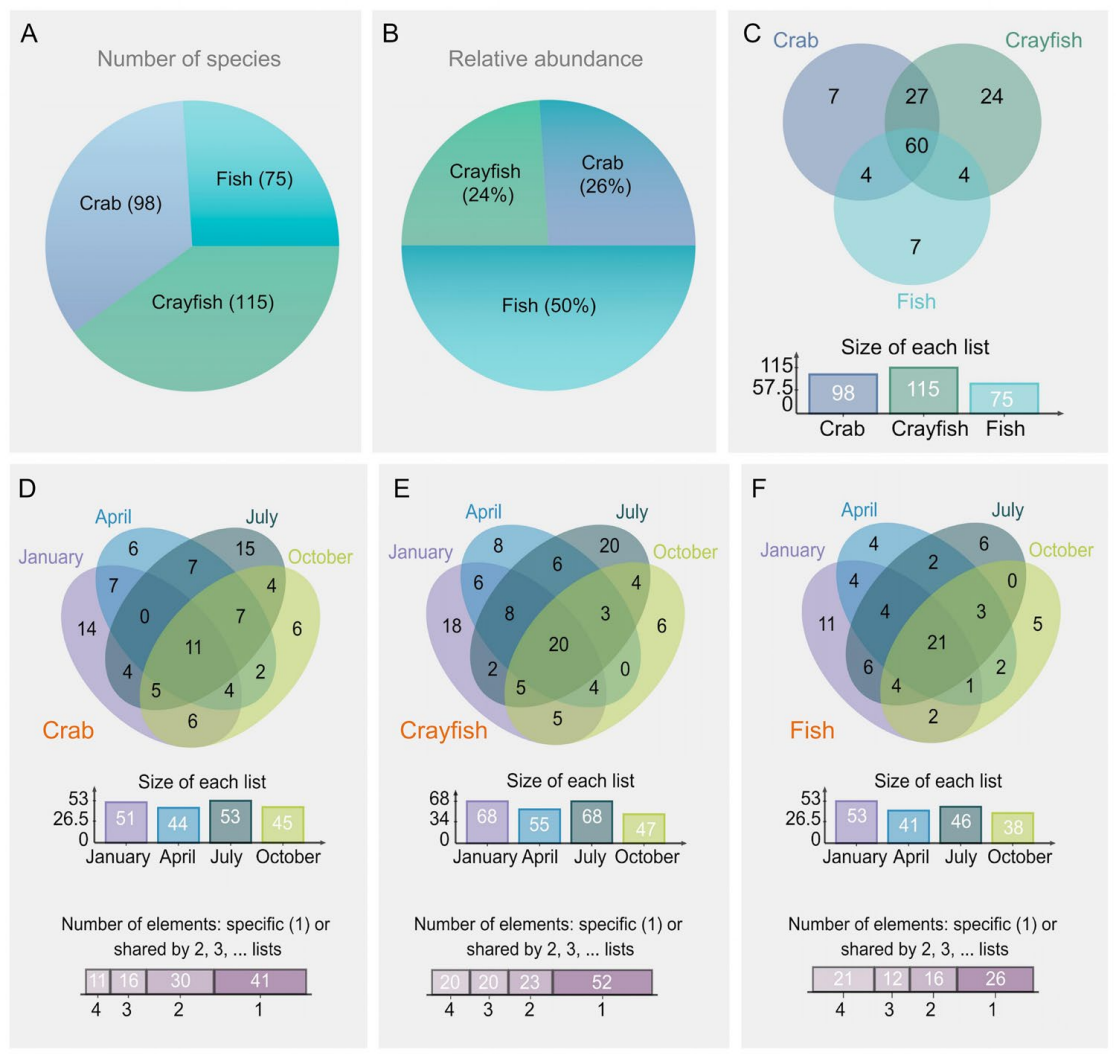
Distribution of zooplankton in three types of aquaculture ponds across different seasons. **A** Total number of zooplankton in each aquaculture pond. **B** Relative abundance of zooplankton in the three types of aquaculture ponds. **C** Shared and unique zooplankton species in the three types of aquaculture ponds. **D**–**F** Seasonal variations in the number of zooplankton in crab (**D**), crayfish (**E**), and fish (**F**) ponds

A total of 132 species of zooplankton were identified based on their morphological characteristics: 61 species of rotifers, 29 species of protists, 28 species of cladocerans, and 14 species of copepods. Under different habitat conditions, the crayfish ponds hosted the largest number of species (115), followed by the crab ponds (98), whereas the fish ponds had the fewest species (75) (Fig. 2A and Table S2). Although the crayfish ponds had the highest species diversity, they exhibited the lowest relative abundance, accounting for only 24% of the total zooplankton abundance. Conversely, the fish ponds, which had the fewest species, showed the highest relative abundance, comprising half of the total zooplankton abundance (Fig. 2B). Most zooplankton species were common across the three types of ponds, suggesting similar patterns of community succession among them (Fig. 2C). The analysis of seasonal variation patterns in different ponds indicated no significant seasonal variation in zooplankton communities among the crab ponds (*X*^2^ = 1.2176, *df* = 3, *P*-value = 0.7488), crayfish ponds (*X*^2^ = 5.395, *df* = 3, *P*-value = 0.1451), and fish ponds (*X*^2^ = 2.8989, *df* = 3, *P*-value = 0.4075; Fig. 2D–F). However, a similar seasonal trend was observed across all three types of ponds, with zooplankton numbers peaking in January and July and decreasing in April and October (Fig. 2D–F).

### Community composition and diversity of zooplankton in different aquaculture ponds across seasons

**Fig. 3:**
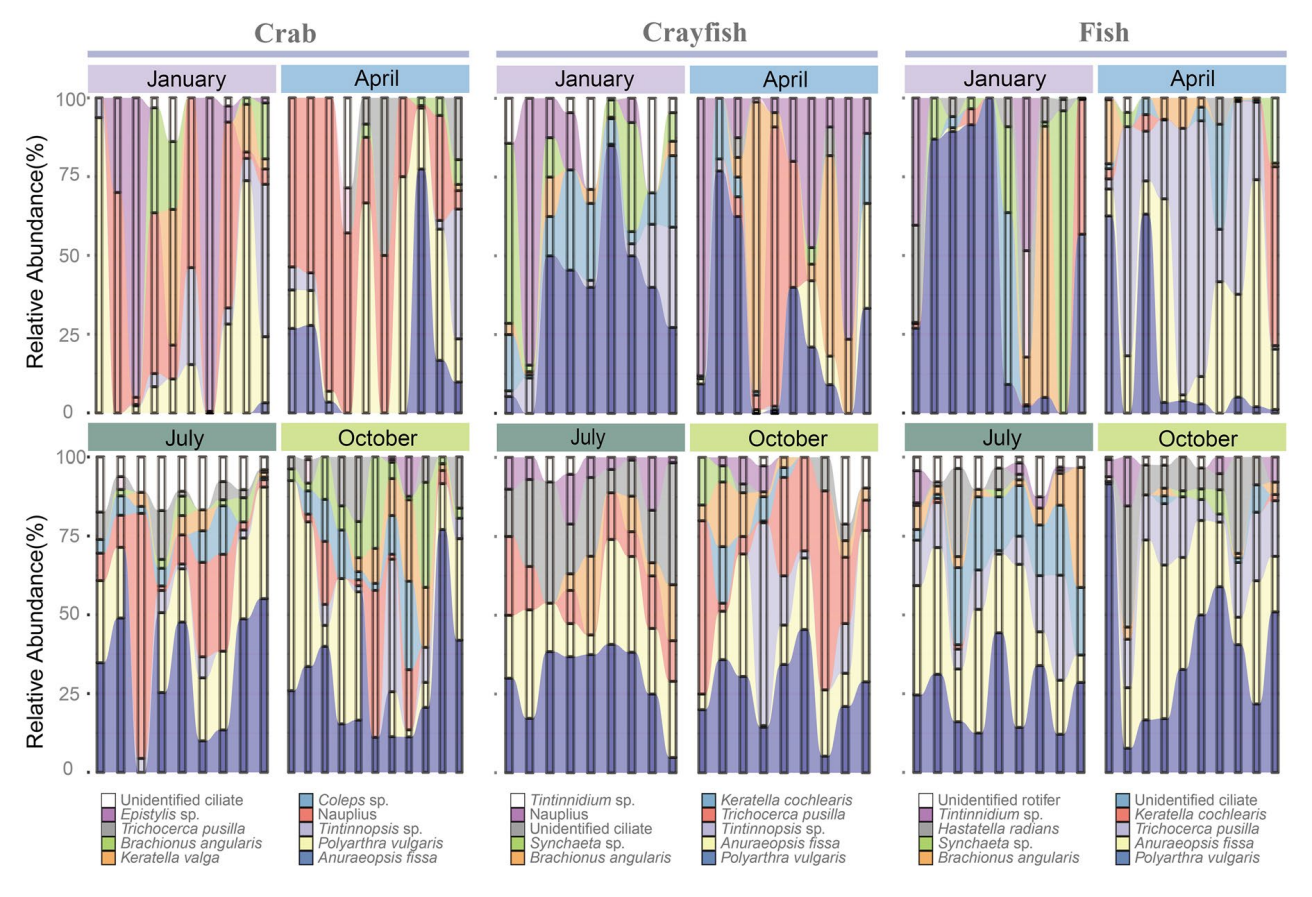
Relative abundance of the top 10 zooplankton taxa in three types of aquaculture ponds

**Fig. 4:**
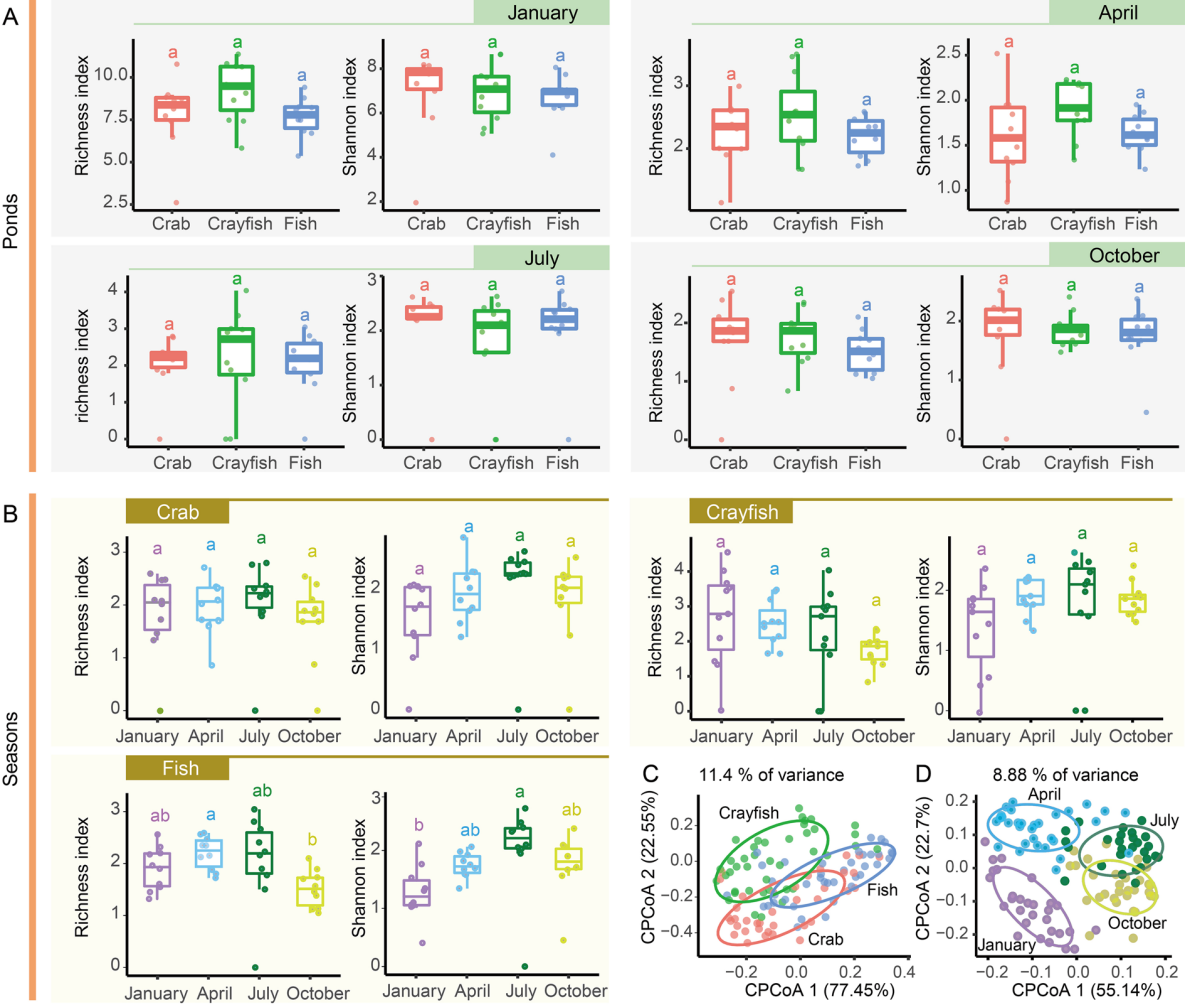
Differences in diversity indices (richness and Shannon) among three types of aquaculture ponds across different seasons. **A** Differences in the richness and Shannon indices in January, April, July, and October. **B** Seasonal diversity differences in crab, crayfish, and fish ponds. **C**, **D** Distribution differences in beta diversity based on the Bray–Curtis distance across different aquaculture ponds (**C**) and seasons (**D**)

Taxonomic analysis revealed that *Polyarthra vulgaris*, *Anuraeopsis fissa*, and *Tintinnopsis* sp. were the dominant species in the zooplankton communities of the crab and crayfish ponds, comprising 48.7% and 44.2% of the communities, respectively. In the fish ponds, the dominant species were *P. vulgaris*, *A. fissa*, and *Trichocerca pusilla*, together accounting for 50.3% of the community (Fig. 3). Notably, the relative abundance of *P. vulgaris* (33.3%) was the highest in the crab ponds compared with the other pond types (Fig. 3). Seasonal patterns of zooplankton communities indicated similar trends for *A. fissa*  across the three pond types, with a relative abundance of nearly zero in January and increasing rapidly from April to October (Fig. 3). *P. vulgaris* maintained a higher relative abundance than other taxa throughout all seasons in all pond types, with the lowest abundance observed in April (Fig. 3). Temporal analysis of diversity showed no significant differences in the richness and Shannon indices or biomass between the crab and crayfish ponds across different seasons (Fig. 4A and Tables S3 and S4). However, in the fish ponds, the richness index differed significantly between April and October, whereas the Shannon index varied significantly between January and July (Fig. 4B and Table S3). In the crayfish ponds, significant differences in biomass were observed between July and January, as well as between October and January (Table S3). CPCoA indicated that zooplankton communities clustered more according to pond type (11.4% variance) than seasonal changes (8.9% variance) (Fig. 4C, D; Table 1). This spatiotemporal distribution pattern was further supported by the Adonis and ANOSIM analysis results (*R*^2^ ≥ 0.154, *P* < 0.001) (Table 1). Seasonal changes had minimal effect on the zooplankton community distribution (*R*^2^ ≤ 0.083, *P* < 0.001) (Table 1). Moreover, the interaction of temporal and spatial factors on the distribution of zooplankton communities (*R*^2^ = 0.280, *P* < 0.001) was greater than the spatial variation alone (Table 1).

**Table 1:** Significance test of the temporal and spatial distributions of zooplankton community structure

Group	Adonis		Anosim	
	*R*^2^	*P*	*R*	*P*
Ponds	0.154	< 0.001	0.292	< 0.001
Seasons	0.046	< 0.001	0.083	< 0.001
^a^S × P	0.280	< 0.001	–	–

^a^S × P, the interactive effects of ponds and seasons

### Driving factors for zooplankton community assembly in aquaculture ponds

**Fig. 5:**
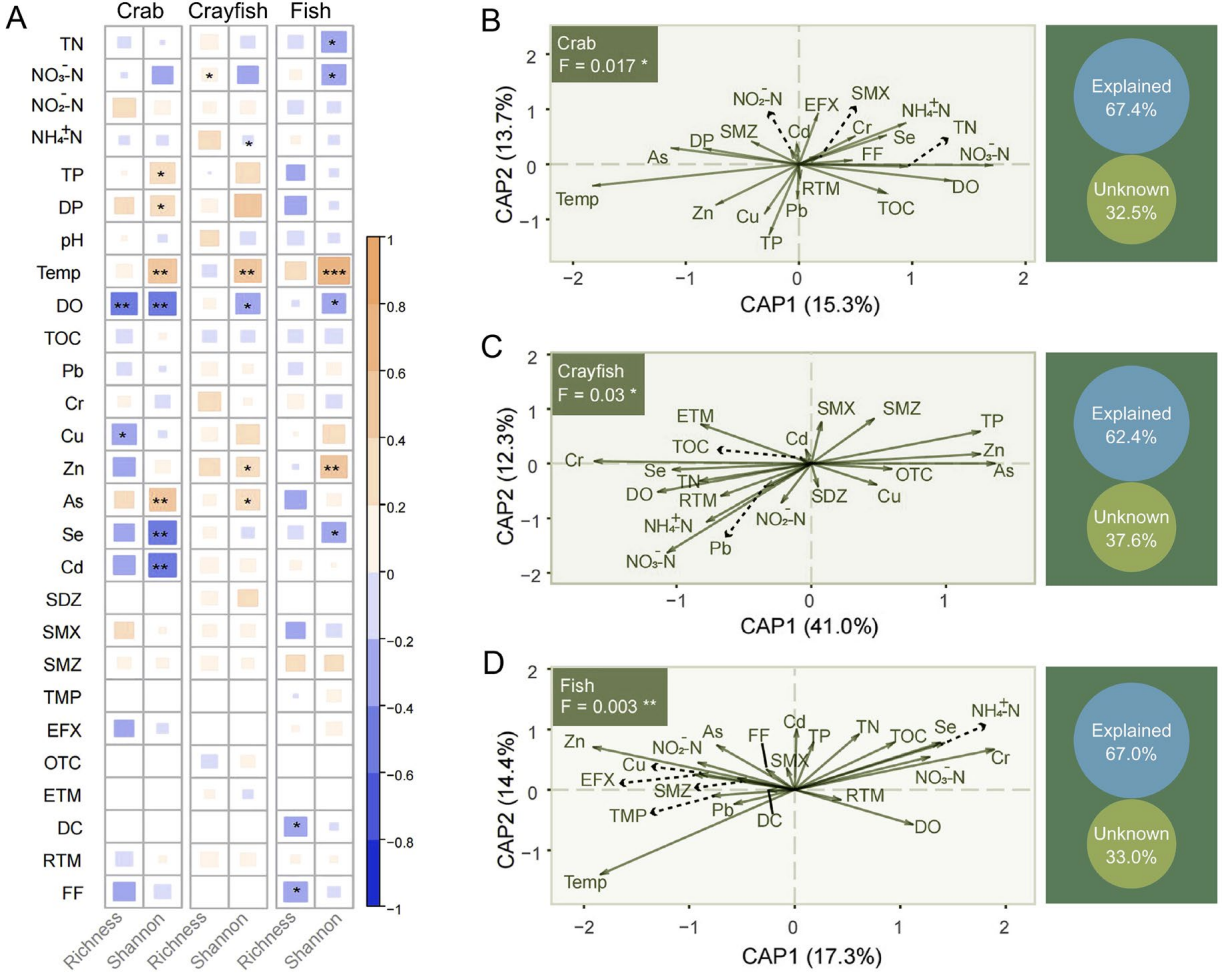
Correlations between zooplankton community diversity and environmental factors in crab, crayfish, and fish ponds. **A** Correlations between alpha diversity (richness and Shannon indices) and environmental factors. **B**–**D** Constrained analysis of principal coordinates (CAP) demonstrating the influence of environmental factors on the distribution of zooplankton communities (*P < 0.05; **P < 0.01; ***P < 0.001)

Correlation analysis revealed that the alpha diversity of zooplankton communities in the crab ponds was significantly influenced by TP, DP, temperature, DO, Cu, Se, As, and Cd (*P* < 0.05). In the crayfish ponds, NO_3_^−^-N, NH_4_^+^-N, temperature, DO, Zn, and As significantly affected the alpha diversity (*P* < 0.05). In the fish ponds, alpha diversity significantly correlated with TN, NO_3_^−^-N, temperature, DO, Zn, Se, DC, and FF (*P* < 0.05) (Fig. 5A and Table S5). Temperature and DO emerged as significant factors influencing alpha diversity across all three pond types (*P* < 0.01). Additionally, antibiotics (DC and FF) significantly impacted the alpha diversity in the fish ponds only (Fig. 5A and Table S5).

**Fig. 6:**
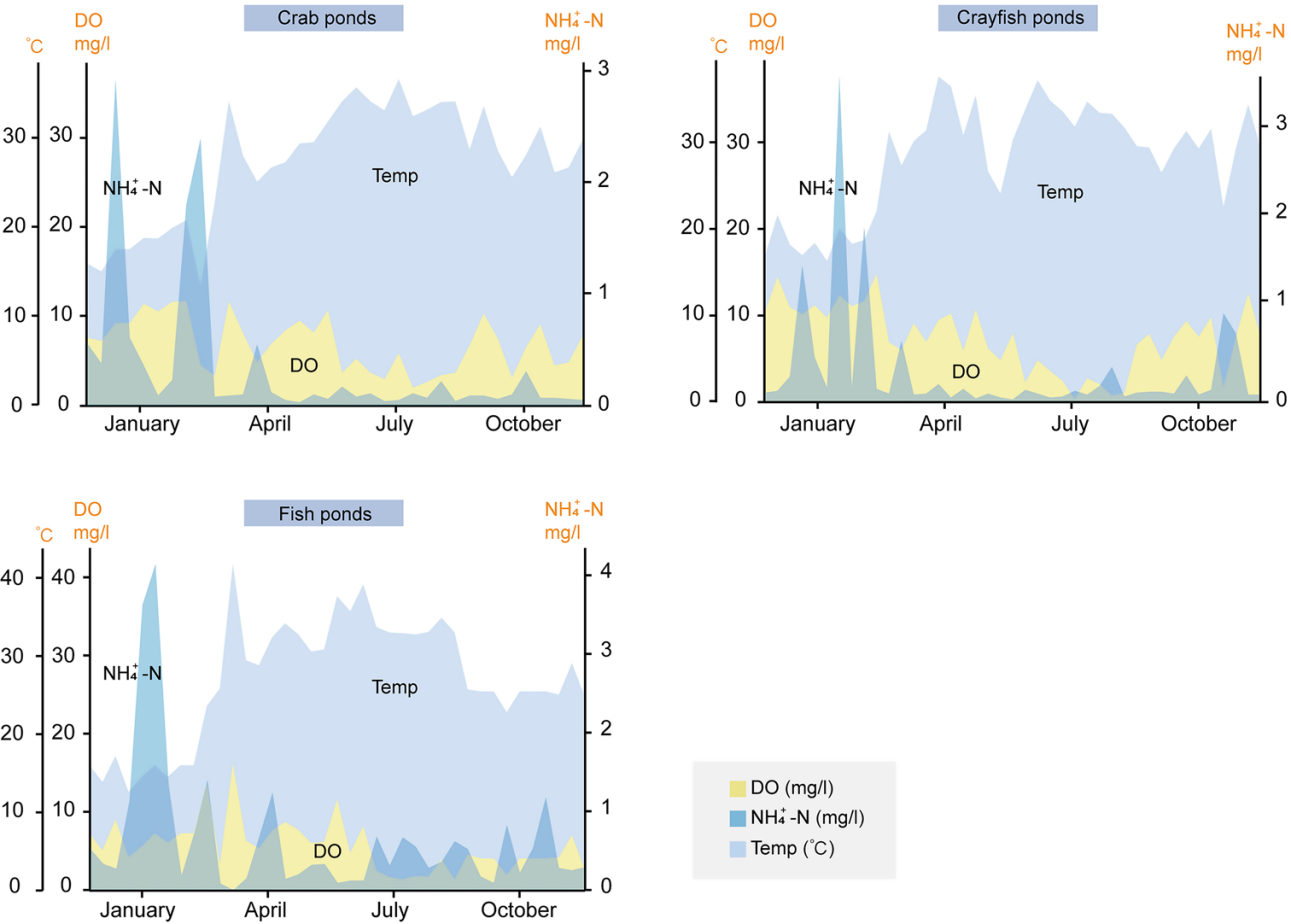
Seasonal variation trends of temperature, dissolved oxygen (DO), and ammonia nitrogen (NH_4_^+^-N) in the three types of aquaculture ponds

The main environmental factors that affected zooplankton beta diversity were identified through CAP. In the crab ponds, TN, NO_3_^−^-N, NH_4_^+^-N, and TP had the greatest observed effects on beta diversity (Fig. 5B and Table S6). In the crayfish ponds, NO_3_^–^-N, Cr, and Zn significantly impacted community distribution (Fig. 5C and Table S7). The fish ponds were influenced by a broader range of environmental variables, including NO_3_^−^-N, NH_4_^+^-N, temperature, DO, TOC, Cr, Zn, and SMZ (Fig. 5D and Table S8). Environmental factors accounted for more than 60% of the community variability, suggesting that deterministic environmental variables play a crucial role in zooplankton assembly (Fig. 5B–D). Meanwhile, the contributions of regular water parameters, nutrients, antibiotics, and heavy metals to the variations in zooplankton beta diversity were evaluated using Mantel and partial Mantel tests. Regular water parameters and nutrients were more predictive of zooplankton community changes than the other factors (Table S9). Random forest analysis revealed that temperature (*P* = 0.01) was the best predictor of zooplankton community variability, followed by N${\text{H}}_{4}^{+}$-N (*P* = 0.03; Table S10). Among all environmental factors, temperature, DO, and N${\text{H}}_{4}^{+}$-N had significant impacts on zooplankton diversity in the three types of aquaculture ponds. These factors exhibited nearly consistent variation patterns across various seasons in all three aquaculture ponds. DO and NH_4_^+^-N levels were higher in January and April, whereas temperatures peaked in July (Fig. 6).

**Fig. 7:**
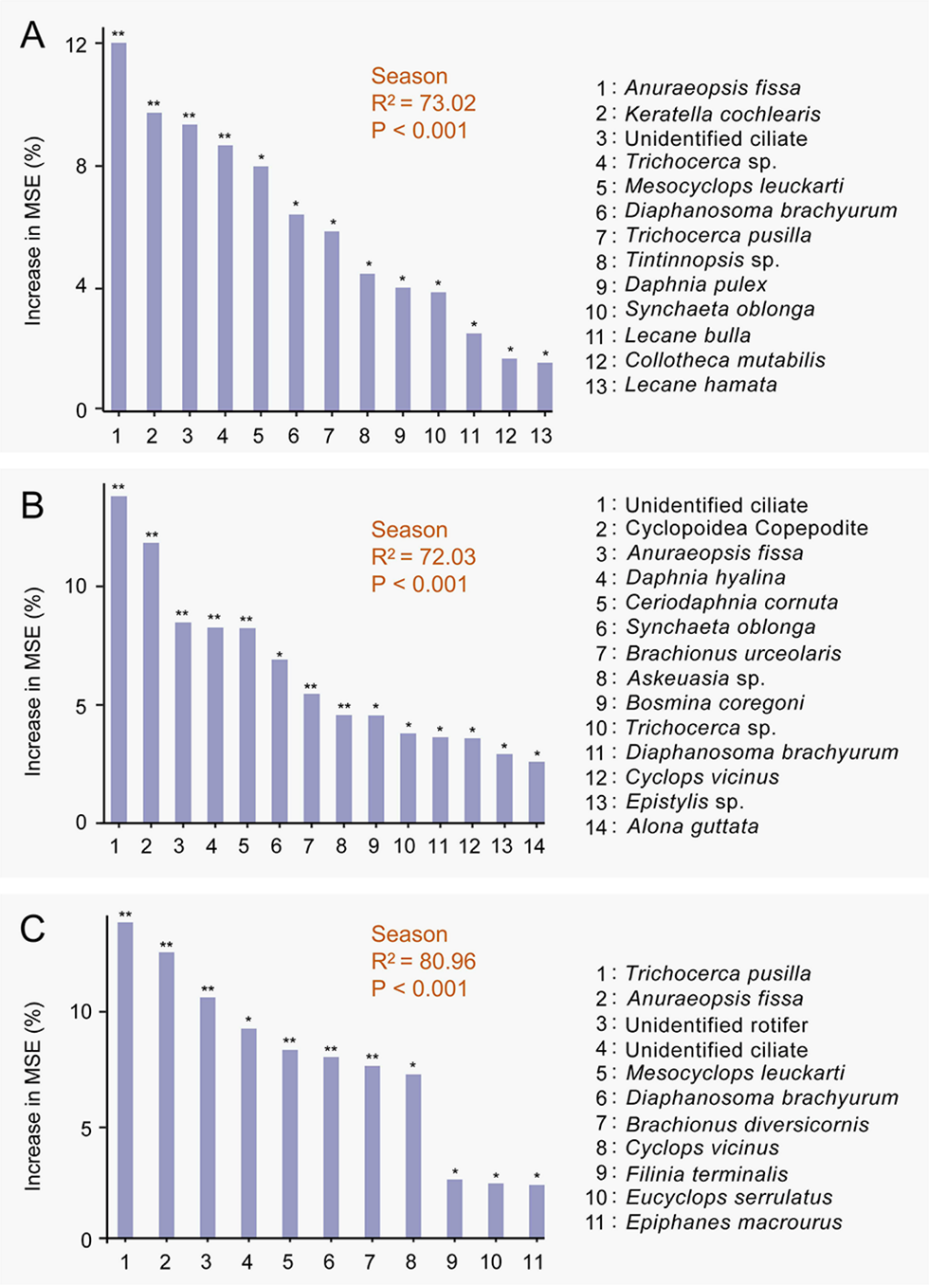
Prediction results of random forests on the relative importance of zooplankton sensitivity to seasonal changes in crab (**A**), crayfish (**B**), and fish ponds (**C**). The variable with increased MSE% is used to estimate the relative importance of species, with higher MSE% values indicating greater importance (MSE: mean squared error; *P < 0.05, **P < 0.01; only the significant species are displayed)

The random forest model was also used to identify species sensitive to seasonal changes. The explanatory power of the three full models exceeded 70% and was statistically significant (*P* < 0.001), indicating model reliability (Fig. 7). In the crab, crayfish, and fish ponds, 13.3%, 12.2%, and 14.7% of the zooplankton species respectively exhibited significant seasonal sensitivity (Fig. 7 and Table S11). While some species, such as *A. fissa*  and *Diaphanosoma brachyurum*, were universally sensitive to seasonal changes across all pond types, each aquaculture pond also hosted its own set of species that were uniquely sensitive to seasonal variation. For instance, *Synchaeta oblonga* and *Trichocerca* sp. were sensitive to seasonal changes in the crab and crayfish ponds but showed no significant seasonal variation in the fish ponds (Fig. 7C and Table S11).

### Analysis of neutral and null community models to evaluate the relative importance of stochastic and deterministic processes

**Fig. 8:**
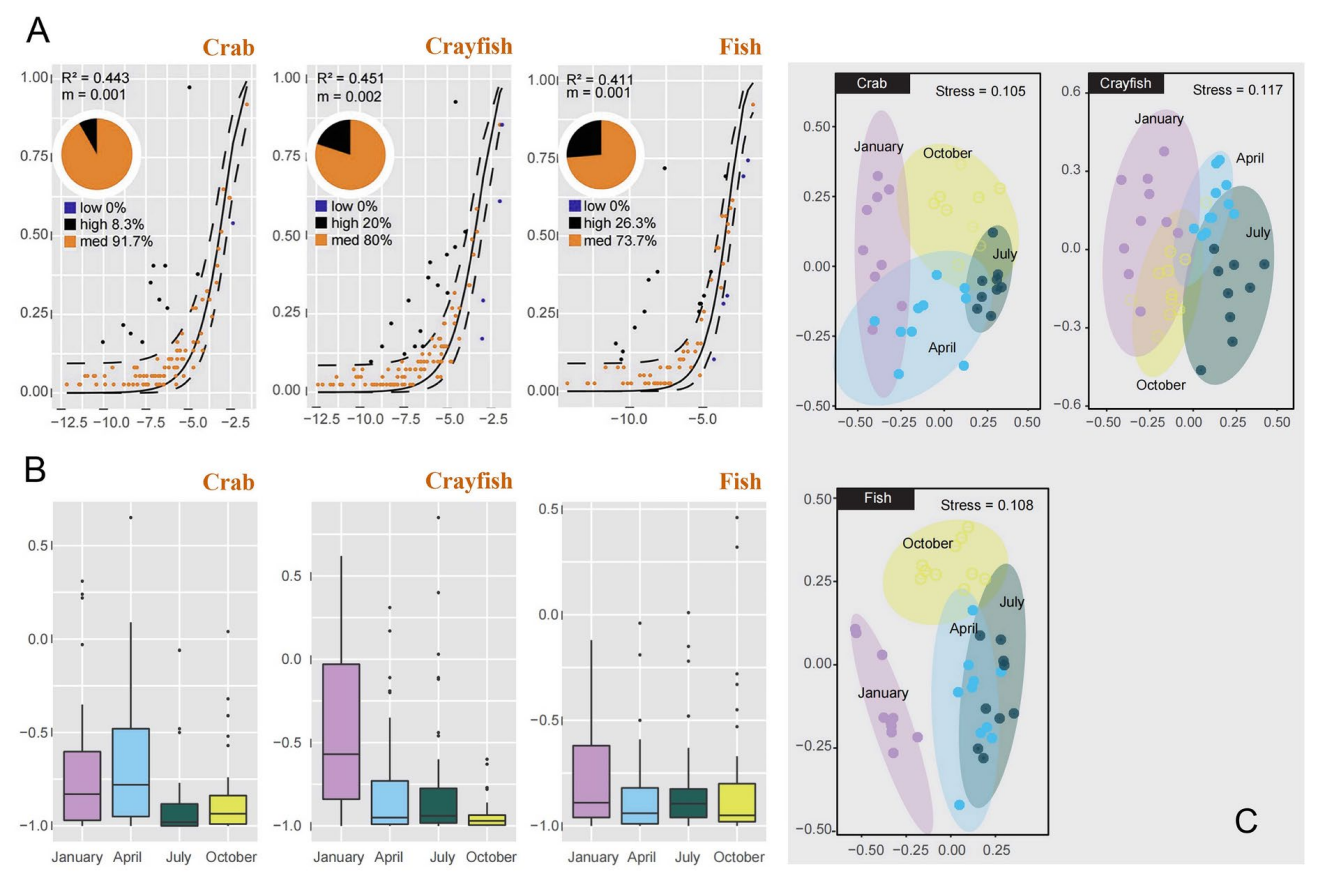
Neutral and null community models assessing the relative importance of stochastic and deterministic processes in zooplankton community assembly among aquaculture ponds. **A** Prediction results of NCM in crayfish, crab, and fish ponds. The solid black lines indicate the best fit for the neutral model, whereas the dashed black lines represent 95% confidence intervals around the model prediction. The pie charts depict the relative abundance of the overrepresented (black), neutrally distributed (orange), and underrepresented species (blue) in each pond. **B** Null model results based on the improved Raup–Crick index for crab, crayfish, and fish ponds across four seasons. Values near 0 indicate no deviation from the null expectation, values near 1 indicate higher similarity than expected, and values near − 1 indicate lower similarity than expected. **C** NMDS based on the βRC index demonstrating community similarity between different seasons in different aquaculture ponds

The neutral community model (NCM) only partially explained the relationship between species occurrence frequencies and their relative abundance, accounting for 44.3%, 45.1%, and 44.1% in the crab, crayfish, and fish ponds, respectively (Fig. 8A). Additionally, the migration rate (“*m*”) exhibited a similar pattern across the three aquaculture ponds, ranging from 0.001 to 0.002 (Fig. 8A). This suggests that certain environmental factors strongly constrained zooplankton dispersal, indicating that deterministic processes predominantly drive zooplankton community assembly in aquaculture ponds.

This finding was further supported by the null model analysis based on the Raup–Crick dissimilarity index. The null model results demonstrated that the Raup–Crick *β* deviation values (βRC) for zooplankton communities tended to deviate from zero and approach − 1 across the three pond types and seasons (Fig. 8B). This indicated that specific ecological factors exerted strict influences on zooplankton community assembly in the different aquaculture ponds. Notably, βRC values were lowest in July, October, and April for the crab, crayfish, and fish ponds, respectively, suggesting slight seasonal variations in the assembly process of zooplankton communities. Moreover, the NMDS analysis based on the Raup–Crick dissimilarity index revealed that community dissimilarity varied with seasons (Fig. 8C). Overall, evidence from both the NCM and null community models suggests that deterministic processes play a major role in the assembly of zooplankton communities in freshwater aquaculture ponds. Although seasonal changes impact zooplankton communities, these changes are secondary to the overriding influence of environmental differences.

## Discussion

### Dominant zooplankton species and their seasonal sensitivity in aquaculture ponds

Zooplankton are vital organisms in aquaculture ponds, serving as key components of aquatic food webs. They transfer energy and matter from primary producers to higher trophic levels and play a significant role in biogeochemical cycling (Karpowicz et al. 2020b; Pong-Masak and Pirzan 2006). Changes in zooplankton biodiversity are crucial for maintaining the health and productivity of aquaculture systems. In this respect, previous studies on zooplankton community changes have often focused on rotifers, cladocerans, and copepods while largely overlooking protists (Brito et al. 2020; Ramzan et al. 2020). However, the importance of protistan predation to zooplankton dynamics has been recognized (Stoecker and Pierson 2019). In this study, we monitored the changes in rotifer, cladoceran, copepod, and protistan communities in three types of aquaculture ponds over a year. We found that the abundance of zooplankton followed this order: rotifers, protists, cladocerans, and copepods. This pattern aligns with findings from previous studies on zooplankton community structures in disturbed wetlands (Shah et al. 2021) and shallow lakes (Cabral et al. 2020). Additionally, we observed that the total zooplankton biomass was higher in fish ponds compared with those in the crab and crayfish ponds (180.10 vs. 111.95 and 100.57 mg/l, respectively), despite the presence of fewer zooplankton species. In July, a notable difference in biomass was observed between the fish and crab ponds (Table S4), which might be attributed to intensified feeding practices in the fish ponds during this period. The increased nutrient input supports phytoplankton growth, indirectly enhancing zooplankton biomass. Although fish exert strong predation pressure, their selective feeding on larger zooplankton allows smaller, rapidly reproducing species to thrive. In contrast, the lower nutrient input and stable predation pressure in the crab ponds likely result in less variation in zooplankton biomass. This pattern reflects the effects of selective predation by fish, as heavy predation can alter zooplankton community structures (Jeppesen et al. 2000; Gyllström et al. 2005), reducing diversity but favoring smaller, fast-reproducing species that contribute to maintaining biomass. 

The rotifer *Polyarthra vulgaris* was the dominant species in the zooplankton communities, aligning with earlier findings (Contreras et al. 2009; Galkovskaya and Mityanina 2005). As a perennial and eurythermal species, *P.* *vulgaris* demonstrates significant environmental adaptability (Shah et al. 2015), which explains its prevalent abundance in aquaculture ponds (Fig. 3). In total, we identified 27 zooplankton species that are sensitive to seasonal changes, nearly half of which are rotifers (Fig. 7 and Table S11). Rotifers, particularly in planktonic environments, are known to exhibit significant seasonal variations (Almeida et al. 2006). For example, *Anuraeopsis fissa*, a common bacterio-detritophagous rotifer with active feeding behavior (Goździejewska et al. 2025), showed significant seasonal associations in all three types of aquaculture ponds. This could be attributed to the pivotal role *A. fissa* plays in the structure of zooplankton communities, which is crucial for the healthy development of aquaculture. Notably, on average, only 13.4% of the zooplankton species identified in each pond type exhibited significant sensitivity to seasonal changes. This suggests that seasonal variation might not be the primary driver of community variation in zooplankton within aquaculture ponds. In these environments, zooplankton communities are primarily shaped by human management practices and stocking densities, differing from natural ecosystems where communities evolve through more complex, long-term ecological processes.

### Environmental determinants of zooplankton diversity in different types of aquaculture ponds

The zooplankton alpha diversity exhibited significant seasonal differences only in the fish ponds, probably because of the top-down control by fish and variations in water quality parameters (Pinelalloul et al. 1995). The distribution of zooplankton communities varied significantly over time and space, particularly in spatial dimensions. This variation is closely related to distinct environmental conditions, with studies indicating that zooplankton distribution can fluctuate greatly among different sampling points (Kamboj and Kamboj 2020). The variations in farming species, stocking density, waste excreta, food residue, and the use of pharmaceutical preparations among the three types of aquaculture ponds significantly influenced microbial communities (Liu et al. 2020b). These factors contributed to the pronounced spatial differences observed, which might explain why zooplankton diversity responded differently to environmental stressors across the different aquaculture types. Moreover, a significant correlation between various heavy metals and zooplankton diversity was observed, likely resulting from the materials used for pond fertilization and the application of lime, which can introduce heavy metals into aquatic ecosystems (Ajewole et al. 2021).

Additionally, a significant impact of antibiotics on zooplankton diversity was observed exclusively in the fish ponds. This result is closely linked to the more frequent and intensive use of antibiotics in fish ponds than in other pond types as part of disease management practices (Chen et al. 2018). Although antibiotics are crucial for controlling pathogens, their unintended consequences on nontarget organisms, such as zooplankton, are frequently overlooked. Antibiotics can disrupt the balance of aquatic ecosystems by affecting zooplankton populations, which are integral to the food web. This disruption may lead to shifts in community composition and a decline in diversity, ultimately impacting ecosystem function and stability. Several studies have shown that antibiotics can alter the growth, reproduction, and survival of zooplankton, which in turn affects their role in nutrient cycling and energy transfer across trophic levels. For instance, Akbar et al. (2020) found that antibiotic exposure affected the life history traits of *Daphnia magna*, hampering its reproduction and survival, particularly under poor dietary conditions. Similarly, Pan et al. (2017) showed that the antibiotic norfloxacin disrupted the relationship between *Scenedesmus quadricauda* and *D. magna*, leading to increased grazing pressure despite reduced grazing activity. Long-term antibiotic exposure can also have multigenerational impacts. In this regard, Cooper et al. (2022) demonstrated that prolonged exposure to antibiotics altered the microbiome of *D. magna*, affecting both its health and fitness. Sathicq et al. (2021) emphasized that meiofauna, including zooplankton, could serve as reservoirs for antibiotic-resistant and potentially pathogenic bacteria, facilitating the spread of resistance in aquatic ecosystems.

In addition, Pei et al. (2024) highlighted that coexposure to antibiotics and microplastics can further affect the ecological roles of zooplankton, thus amplifying the potential risks to ecosystem stability. Furthermore, Perera et al. (2022) discussed how zooplankton can act as carriers of pathogenic bacteria, facilitating their survival and spread in aquatic environments, which may exacerbate the risks associated with antibiotic use. The more frequent antibiotic applications in the fish ponds likely explain why the impact on zooplankton diversity was significant only in these ponds. Although numerous environmental factors influenced the zooplankton community diversity, temperature and DO were consistently associated with zooplankton alpha diversity across all three pond types. Previous research has emphasized the close relationship between zooplankton community structure and oxygen stress (Karpowicz et al. 2020a), as well as the significant effects of electrical conductivity, salinity, and water temperature (Abdallah et al. 2017). Previously, Roman et al. (2012) demonstrated that hypoxia specifically affects zooplankton distribution.

Furthermore, our findings indicate that temperature and ammonia nitrogen exert significant influences on zooplankton community diversity in aquaculture ponds. Temperature has long been recognized as a critical environmental factor shaping zooplankton communities, with numerous studies in aquaculture systems underscoring its importance in shaping zooplankton composition. For instance, in aquaculture ponds, higher water temperatures have been shown to promote the growth of certain species, such as rotifers and cladocerans, while decreasing the abundance of others (Musa et al. 2021). Similarly, Coman et al. (2006) reported that water temperature significantly affects zooplankton density and composition, further emphasizing its role in controlling zooplankton diversity in aquaculture systems. Meanwhile, ammonia nitrogen, a key nutrient in aquaculture environments, has also been identified as a major factor influencing zooplankton diversity. Elevated ammonia concentrations can lead to reduced zooplankton biomass, highlighting its critical role in shaping zooplankton communities in controlled aquaculture systems (Pimentel et al. 2025). These findings, together with our results, further emphasize the importance of both temperature and ammonia nitrogen in driving zooplankton diversity in aquaculture ponds. 

### Deterministic processes of zooplankton community assembly

The mechanisms that govern community diversity, succession, and biogeography are a core topic in ecology (Zhou and Ning 2017). Nevertheless, they remain particularly poorly understood in zooplankton communities. Most studies on zooplankton have primarily focused on community composition and responses to environmental factors (Blanco-Bercial 2020; Karpowicz et al. 2020a; Kovalev et al. 1999). Only a few have explored the underlying mechanisms driving changes in zooplankton communities. Despite many researchers having examined assembly processes in microeukaryotes (Liu et al. 2020a; Vass et al. 2020; Zheng et al. 2024), zooplankton communities have often been overlooked.

Deterministic and stochastic processes are the primary drivers of community assembly in aquatic ecosystems (Vass et al. 2020; Zhou and Ning 2017). In our study, we combined neutral and null community models based on the Raup–Crick dissimilarity index, as refined by Chase et al. (2011), to evaluate the assembly processes of zooplankton communities in aquaculture ponds (Sloan et al. 2006). Our results indicated deterministic processes as the dominant force shaping zooplankton community assembly. Increasing evidence supports the role of deterministic processes in zooplankton community assembly, particularly in managed aquatic ecosystems and human-impacted water bodies, such as seasonal floodplain–river wetland habitats and polluted semiclosed seas (Corline et al. 2021; Zhao et al. 2021). Studies on microeukaryotic community assembly also align with our findings, suggesting that deterministic processes may be more prominent in water diversion systems (Wei et al. 2024) and in the later stages of aquaculture (Zheng et al. 2024). However, other research suggests that stochastic processes dominate in subtropical rivers (Chen et al. 2019), which contrasts with our findings. This discrepancy may be due to differences in environmental conditions, such as the relatively low levels of disturbance in subtropical rivers.

As fundamentally contrasting mechanisms, stochastic and deterministic processes are driven by different factors. Deterministic processes are shaped by local environmental conditions and biotic interactions, whereas stochastic processes are influenced by random events, such as birth, death, speciation, and immigration (Zhou and Ning 2017). In line with our findings, many studies have highlighted the role of deterministic factors in zooplankton community assembly, such as water residence time and key physicochemical factors (e.g., water temperature and ammonia nitrogen) (Obertegger and Flaim 2018; Pimentel et al. 2025). Evolutionary dynamics also play an important role in shaping community assembly (Pantel et al. 2015). Furthermore, factors such as oxygen concentration, water hardness, conductivity, phosphorus, and unionized ammonia significantly affect zooplankton community structures, with groups like Rotifera and Cladocera being particularly sensitive (Dulić et al. 2014). Additionally, the resuspension of bottom sediments by grass carp significantly alters protist metacommunities in aquaculture ponds (Zheng et al. 2021).

Changes in zooplankton populations can trigger cascading effects throughout the food web, impacting aquaculture species and other higher trophic levels (Kerfoot and DeAngelis 1989; Lomartire et al. 2021). Therefore, managing zooplankton succession by adjusting temperature and ammonia nitrogen levels to utilize zooplankton as natural feed instead of traditional feed for aquaculture species presents a valuable yet challenging task. This balance requires careful management of environmental variables to ensure suitability for aquaculture species (Das et al. 2012). Our research provides key evidence for the sustainable development of aquaculture, highlighting the importance of integrating environmental management with aquaculture practice.

## Conclusion

In this study, we monitored the changes in zooplankton communities, including rotifers, protists, cladocerans, and copepods, across three different freshwater aquaculture ponds over a year, revealing distinct seasonal variations. Our results showed that species richness and biomass exhibited contrasting patterns in crayfish and fish ponds. Although the alpha diversity of zooplankton communities was similar across the three pond types, the beta diversity revealed significantly different spatiotemporal distributions. The alpha diversity of zooplankton in the ponds was influenced by various environmental factors, with temperature and DO being particularly significant across all pond types. Additionally, antibiotics were significantly associated with alpha diversity only in the fish ponds, highlighting the need for the careful management of antibiotic use in these environments to mitigate risks to biodiversity and ecosystem health. Community assembly analysis indicated that deterministic processes, driven primarily by temperature and ammonia nitrogen, governed the zooplankton community assembly across the aquaculture ponds. These findings provide a deeper understanding of the environmental factors influencing zooplankton community dynamics and underscore the critical importance of targeted environmental management practices to enhance zooplankton stability and promote overall aquaculture sustainability.

## Supplementary Information

Below is the link to the electronic supplementary material.

Supplementary file1 (XLSX 75 KB)

## Data Availability

Data will be made available on request.
